# Postural control through force plate measurements in female AIS patients compared to their able-bodied peers

**DOI:** 10.1038/s41598-022-17597-y

**Published:** 2022-08-01

**Authors:** Elżbieta Piątek-Krzywicka, Dorota Borzucka, Michał Kuczyński

**Affiliations:** 1 Faculty of Physiotherapy, University School of Physical Education in Wroclaw, ul. Paderewskiego 35, 51-612 Wrocław, Poland; 2 Faculty of Physical Education and Physiotherapy, Opole University of Technology, ul. Prószkowska 76, 45-758 Opole, Poland

**Keywords:** Motor control, Somatosensory system, Visual system, Musculoskeletal system

## Abstract

The present understanding of the mechanisms responsible for postural deficit in adolescent idiopathic scoliosis (AIS) is still insufficient. This is important because some authors see one of the causes of this disease in the impaired postural control. Moreover, there is a reciprocal link between the level of postural imbalance and the clinical picture of these people. Therefore, we compared the center-of-pressure (COP) indices of 24 patients with AIS to 48 controls (CON) during four 20-s quiet stance trials with eyes open (EO) or closed (EC) and on firm or foam surface. This included sway amplitude, speed, sample entropy and fractal dimension. AIS had poorer postural steadiness only in the most difficult trial. In the remaining trials, AIS did as well as CON, while presenting a greater COP entropy than CON. Thus, the factor that made both groups perform equally could be the increased sway irregularity in AIS, which is often linked to higher automaticity and lower attention involvement in balance control. After changing the surface from hard to foam, puzzling changes in sway fractality were revealed. The patients decreased the fractal dimension in the sagittal plane identically to the CON in the frontal plane. This may suggest some problems with the perception of body axes in patients and reveals a hitherto unknown cause of their balance deficit.

## Introduction

Adolescent idiopathic scoliosis (AIS) is a three-plane deformity of the spine^1^. It is the most common type of scoliosis, affecting 2–4% of adolescents, mainly involving children between 10 and 18 years of age^2^. The severe deformation of body morphology resulting from scoliosis could influence the postural control. This three-dimensional spinal deformity often alters the orientations of the head, trunk, and pelvis in all anatomical planes, as well as resulting in vestibular and trunk muscle imbalance, and proprioceptive disorders, leading to a postural control problem^3^.

The underlying cause of impaired postural control is considered multifactorial. Research in the recent decade has attempted to evaluate if and to what extent AIS patients show proprioceptive and somatosensory impairment which influence on the abnormal function of balance control. It has been indicated that patients with AIS show postural stability deficits/reduced balance control^4,5^. Some evidence suggests that dysfunction of the vestibular system may be a contributing factor to the development of AIS^6^. Asymmetric vestibular function and the related descending drive to the spine musculature can lead to inappropriate trunk muscle activity, spine deformation, and greater instability^7^. On the other hand, several studies on adolescent patients with idiopathic scoliosis revealed their fair postural stability compared to the control group^8^.

There is still too little research in the literature that would examine the posture control system in relation to healthy. Our previous work dealt with how girls with AIS do during active self-correction (ASC) and the possibility of scoliosis impact on posture control^9,10^. Results seem to account for the desirable changes in postural control that were generated by learned ASC movements. In particular, they reflect the tendency of individuals with AIS to optimize their gravity line alignment and their adequate resources of available postural strategies, which are necessary to cope with novel postural challenges. However, there was always something missing and the interpretations were ambiguous.

Hence, this study aimed to compare postural control in girls with AIS with a control group of healthy peers to reveal possible differences in postural strategies between these two groups when subjected to sensory manipulations. A better understanding of these postural strategies in patients with scoliosis will help to plan future studies in a more effective way.

## Materials and methods

This study was approved by the Senate Research Ethics Committee at the University School of Physical Education in Wroclaw, Poland (approval number: 35/2016). Written informed consent was obtained from all participants and their parent(s) or legal guardian(s) prior to their participation. The study goals, procedures, and methods were explained in full, and the subjects were informed that they could withdraw at any time. All methods were carried out in accordance with relevant guidelines and regulations.

### Participants

The postural control system was assessed in 72 subjects: the study group of 24 patients with adolescent idiopathic scoliosis (AIS) from a local therapeutic rehabilitation center and a control group (CON) of 48 healthy adolescents. The biometric characteristics of the subjects including scoliotic curvature details in AIS are presented in Table 1.

**Table 1:** Characteristics of the participants (mean ± SD).

	Study group (AIS) (n = 24)	Control group (CON) (n = 48)
Age (years)	13.4 ± 1.6	12.7 ± 1.4 (p = 0.17)
Height (m)	159.5 ± 10.1	161.4 ± 9.1 (p = 0.40)
Body mass (kg)	50.8 ± 7.8	52.4 ± 11.6 (p = 0.53)
AIS curve pattern	75% R thoracic/L lumbar 25% L thoracolumbar	–
Primary Cobb angle (°)	24.5 ± 7.5	–
Risser sign	2.8 ± 0.8	–

The p-values are the results of between-group comparison of the respective data (*t* test).

Inclusion criteria to the study group were diagnosis of AIS by an independent physician based on the current AP X-rays in upright position and receiving conservative treatment in the form of physiotherapeutic scoliosis-specific exercise (PSSE) for at least 3 months. Female gender was an additional inclusion criterion for both groups.

Exclusion criteria both for the study and control group were subjects with a history of spine surgery, musculoskeletal or neurological diseases and with lower limb abbreviations. Moreover, subjects with any posture defects and scoliosis were also excluded from the control group. All subjects had normal or corrected to normal vision.

### Methods

Postural control was assessed on a Kistler force platform (Kistler 9281CA, Winterthur, Switzerland). The study protocol was the same as in Piątek et al.^10^. Two-dimensional horizontal coordinates of the COP data were recorded for 20 s at a sampling frequency of 100 Hz.

Each participant performed four quiet standing trials: on a firm or foam surface with eyes open or closed conditions. These included standing upright in a neutral and comfortable stance with the arms relaxed at the sides. The trial order was randomized. The participants were instructed to stand as motionless as possible. The feet position (5 cm apart) was standardized on the surface to ensure repeatability across trials and participants. Data acquisition began when the subject signaled that they were ready^10^.

The COP data may be represented and analysed as planar trajectories (stabilograms) which have a bivariate distribution, jointly defined by the medial–lateral (ML) and anterior–posterior (AP) coordinates or as one dimensional time-series in the ML and AP axes separately^11^. On the basis of the COP recordings the following COP parameters were computed:

1. The COP mean radius (mm)—the mean distance of all successive values of the stabilogram in the ML/AP plane from the mean COP.
2. The COP ML and AP variability (mm)—standard deviation of COP displacements.
3. The COP ML and AP mean speed (mm/s)—the COP path length divided by trial duration; this scalar index is often incorrectly referred to as the COP velocity, which is a vector quantity.
4. The COP ML and AP sample entropy (–)—a non-linear dynamic COP parameter, the higher values of which indicate greater COP irregularity and may often indicate smaller attentional resources devoted to maintaining the balance (greater automaticity).
5. COP fractal dimension (–)—a non-linear dynamic parameter of COP, the higher values of which indicate greater COP complexity and may often imply better adaptation to environmental demands. Detailed information on the calculation of the last two COP parameters can be found in Bieć et al.^12^.

### Statistics

The Statistica 12.0 software package (StatSoft, Tulsa, OK, USA) was used to carry out all statistical analyses. The data met the criteria of the normal distribution for all variables of interest. All between-group differences for identical stance conditions were investigated by means of planned comparisons. To find the potential effects of the stance conditions on changes of the COP indices, each dependent variable except the stabilogram mean radius was subjected to four-way analysis of variance (Anova). This included 2 groups (AIS and CON) × 2 surfaces (firm and foam) × 2 visual conditions (EO = eyes open and EC = eyes closed) × 2 planes (AP and ML) Anova with repeated measures on the last three factors. The stabilogram mean radius as a bidirectional measure was subjected to three-way Anova (without the plane factor). Selected pairwise comparisons were explored using Tukey test. The level of significance was set at p < 0.05.

## Results

### General results

The initial Anova produced a number of overall results that did not differentiate between the groups being compared. Although not directly related to the purpose of this study, they seem helpful in interpreting those observations (see specific results) where intergroup differences were revealed. Their additional importance is that they justify the next steps in the Anova procedure. Furthermore, they provide a good baseline obtained from a total of 72 female participants that illustrates the effects of surface, vision, and plane on all stabilographic parameters used. The following main effects were observed:

1. Surface on: radius (F(1,70) = 401.01, p < 0.00001), amplitude (F(1,70) = 463.47, p < 0.00001), speed (F(1,70) = 344.04, p < 0.00001), entropy (F(1,70) = 260.97, p < 0.00001), and fractal dimension (F(1,70) = 34.72, p < 0.00001).
  Transition from hard to foam surface increased values of the COP radius, amplitude and speed while decreased the COP entropy and fractality.
2. Vision on: radius (F(1,70) = 179.18, p < 0.00001), amplitude (F(1,70) = 184.21, p < 0.00001), speed (F(1,70) = 257.32, p < 0.00001), entropy (F(1,70) = 40.66, p < 0.00001), and fractal dimension (F(1,70) = 10.61, p = 0.0017).
  Eyes closure increased values of all parameters except entropy which decreased.
3. Plane on: amplitude (F(1,70) = 100.15, p < 0.00001), speed (F(1,70) = 277.55, p < 0.00001), entropy (F(1,70) = 9.26, p = 0.0033), and fractal dimension (F(1,70) = 8.31, p = 0.0052).
  The observed values of COP parameters were higher in AP than ML plane for COP amplitude, speed and entropy. Only sway fractality was higher in the ML plane.

In addition, the following interactions common to both groups were identified.

4. Surface × Vision on: radius (F(1,70) = 78.22, p < 0.00001), amplitude (F(1,70) = 103.55, p < 0.00001), speed (F(1,70) = 176.67, p < 0.00001), entropy (F(1,70) = 4.20, p = 0.045), and fractal dimension (F(1,70) = 7.37, p = 0.0084).
  Standing on foam surface increased COP radius, amplitude and speed much more with eyes closed than with eyes open. Standing on foam reduced entropy more with eyes closed and fractals more with eyes open.
5. Vision × Plane on COP speed (F(1,70) = 30.29, p < 0.00001) indicated that eyes closure increased COP speed to a larger extent with eyes closed than open.

### Specific results

Our investigated groups were not different in terms of age, height and body mass (Table 1). Therefore, these characteristics should not have affected the calculated inter-group differences in calculated sway parameters. The following parameters revealed the influence of Group on their values, usually in interaction with independent variables:

### Sway radius

**Figure 1:**
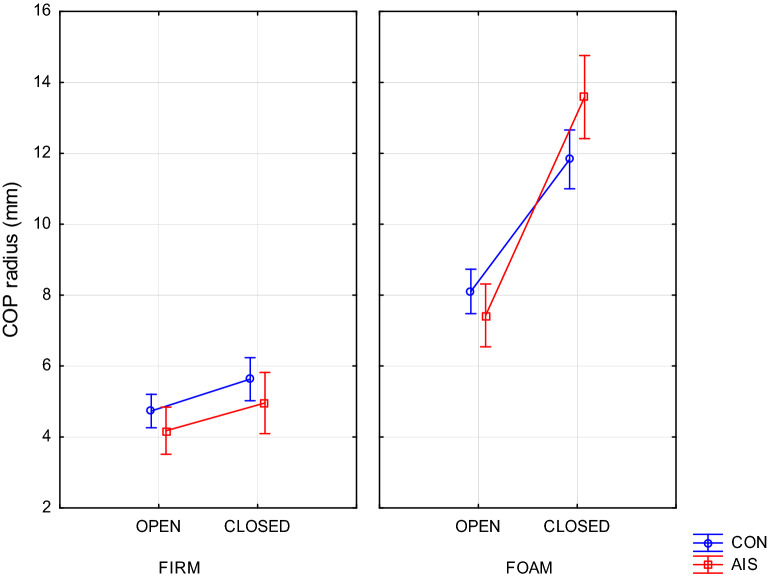
Group × Surface × Plane interaction (p = 0.0076) on COP radius for girls with scoliosis (AIS) and controls (CON) in trials with eyes open or closed on firm or foam surface.

There were two simple interactions: Group × Surface (F(1,70) = 4.63, p = 0.035) and Group × Vision (F(1,70) = 7.18, p = 0.0092) which indicated that foam surface and eyes closure, respectively, increased sway radius more in AIS than in the control group. Additionally, a three-way interaction Group × Surface × Vision (F(1,70) = 7.56, p = 0.0076) showed (Fig. 1) that it was only during the most difficult trial (i.e. with eyes closed on foam surface) when AIS were inferior to controls (p = 0.030).

### Sway amplitude

There were two interactions: Group × Vision (F(1,70) = 7.46, p = 0.0080) and Group × Surface × Vision (F(1,70) = 8.00, p = 0.0061). The former interaction revealed larger increase in sway amplitude caused by eyes closure in AIS than in CON group. The latter one specified this observation as relating only to trials on foam surface, i.e. in more difficult condition.

### Sway mean speed

There were also two interactions: Group × Vision (F(1,70) = 7.99, p = 0.0061) and Group × Surface × Vision (F(1,70) = 9.94, p = 0.0024). In a very similar way as for sway amplitude, the former interaction revealed larger increase in sway mean speed caused by eyes closure in AIS than in CONT group. The latter one specified this observation as relating only to trials on foam surface, i.e. in more difficult condition.

**Figure 2:**
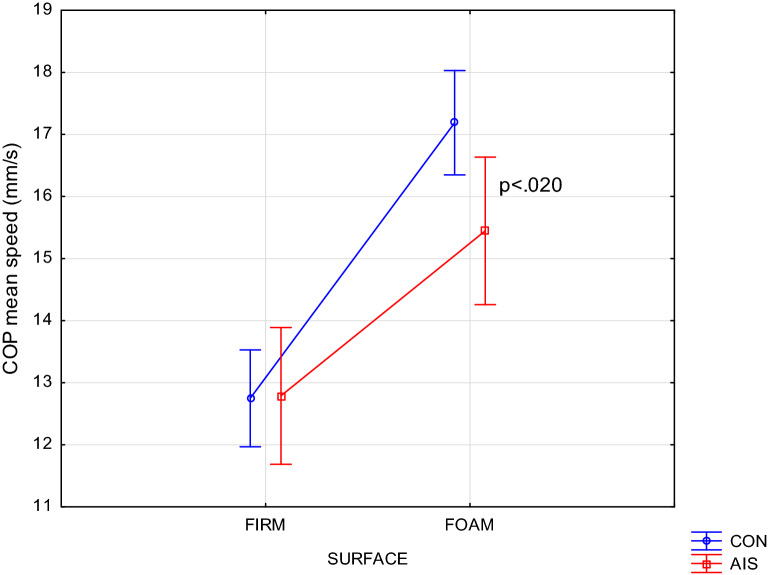
Group × Surface interaction (p = 0.0041) on COP mean speed in AP plane while standing with eyes open.

Interestingly, the same interaction computed in the AP plane only (F(1,70) = 7.02, p = 0.0099) exhibited differences between both groups which depended on visual condition. With eyes closed the advantage of CON over AIS was similar as before. However, with eyes open, it was the AIS group that outperformed controls on foam surface (p = 0.020), as documented by the Group × Surface interaction (F(1,70) = 8.80, p = 0.0041) computed for the left panel in Fig. 2.

### Sway sample entropy

**Figure 3:**
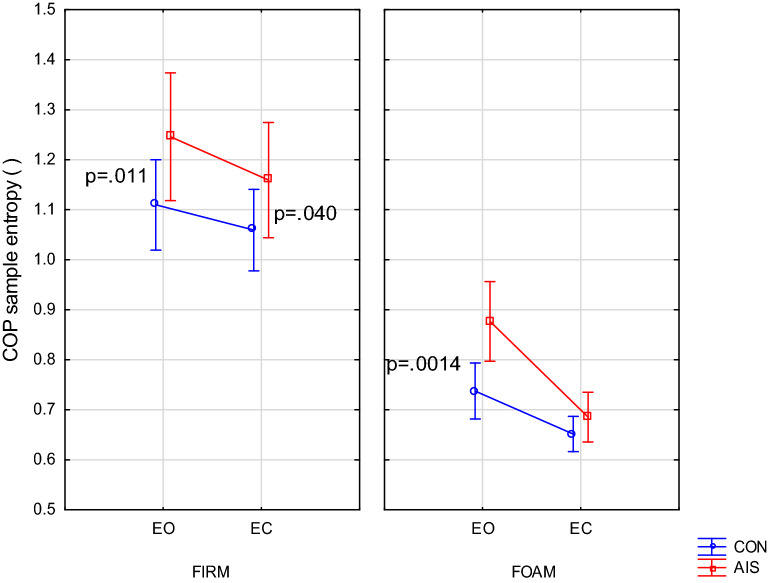
COP sample entropy comparison between girls with scoliosis (AIS) and controls (CON) in trials with eyes open (EO) or closed (EC) on firm or foam surface.

Sway entropy was the only COP parameter that revealed intergroup differences at the main effects level. This main effect of Group (F(1,70) = 9.60, p = 0.0028) showed higher sway irregularity in AIS than in CON. There was also the Group × Vision interaction (F(1,70) = 4.24, p = 0.043) indicating that closing the eyes reduced sway entropy to a greater extent in the AIS group than in CON. Further analysis of this parameter using planned comparisons indicated that it had higher values in AIS in the three easier trials. Only the most difficult trial decreased entropy in AIS to values similar to the control group (Fig. 3).

### Sway fractal dimension

**Figure 4:**
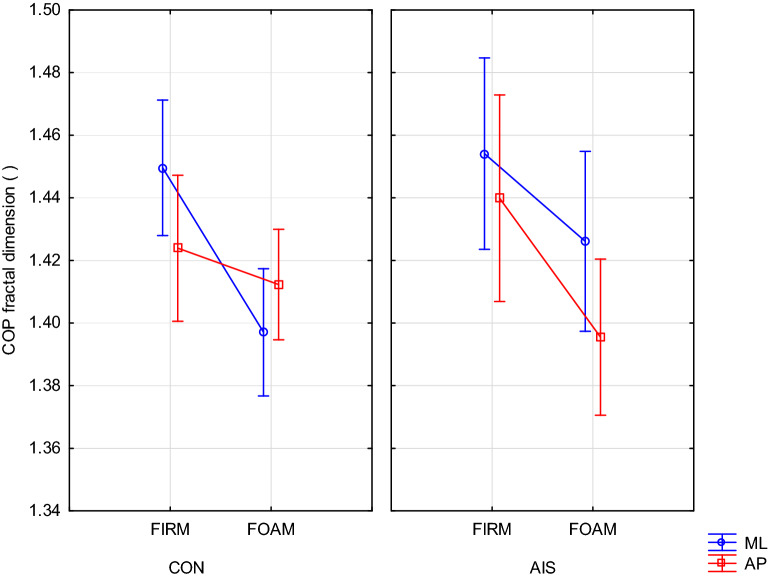
Group × Surface × Plane interaction (p = 0.00079) on COP fractal dimension for girls with scoliosis (AIS) and controls (CON) in trials with eyes open on firm or foam surface in the medial–lateral (ML) and antero-posterior (AP) plane.

We observed one interaction that included a Group factor, namely a strong Group × Surface × Plane interaction (F(1,70) = 12.32, p = 0.00079). This interaction is illustrated in Fig. 4. While the plots in both panels appear similar, the strong interaction is due to the fact that the plots for the AP and ML planes in the right panel are very similar to the plots in the left panel, but in reverse order: ML and AP.

Detailed data for both groups of subjects in all stance conditions are provided in Table 2.

**Table 2:** Mean values (SD) of the COP parameters for two groups (AIS and CONTROL) standing on Firm or Foam surface with eyes open (EO) or closed (EC) in the sagittal (AP) and frontal (ML) plane.

AIS				Control			
Firm		Foam		Firm		Foam	
EO	EC	EO	EC	EO	EC	EO	EC
**Mean radius (mm)**							
4.18 (1.56)^˄^	4.96 (1.64)^˄^	7.43 (2.42)^ǂ^	13.59 (3.42)*	4.73 (1.67)^˄^	5.63 (2.31)^˄^	8.11 (2.04)^ǂ^	11.83 (2.57)
**Amplitude M/L (mm)**							
2.79 (1.15)^˄^	2.24 (1.43)^˄^	5.44 (2.57)^ǂ^	9.58 (2.67)	3.02 (1.24)^˄^	3.31 (1.36)^˄^	5.71 (1.49)^ǂ^	8.54 (2.25)
**Amplitude A/P (mm)**							
3.75 (1.80)^˄^	4.57 (1.94)^˄^	6.05 (1.87)^ǂ^	11.87 (3.22)	4.44 (1.75)^˄^	5.42 (2.42)^˄^	7.13 (2.44)^ǂ^	10.49 (2.60)
**Mean speed M/L (mm/s)**							
9.15 (2.42)^˄^	10.17 (2.67)^˄^	13.56 (3.15)^ǂ^	27.57 (9.21)*	9.24 (2.43)^˄^	10.30 (3.16)^˄^	13.73 (2.73)^ǂ^	22.45 (6.54)
**Mean speed A/P (mm/s)**							
12.79 (2.95)	14.89 (4.09)^˄^	15.45 (3.36)*^ǂ^	32.70 (10.9)	12.75 (2.57)^˄^	15.06 (3.64)^˄^	17.19 (2.68)^ǂ^	29.18 (7.85)
**Entropy M/L ( )**							
1.25 (0.35)^˄^	1.16 (0.31)^˄^	0.88 (0.25)*	0.69 (0.12)	1.11 (0.29)^˄^	1.06 (0.27)^˄^	0.74 (0.16)	0.65 (0.13)
**Entropy A/P ( )**							
1.34 (0.38)*^˄^	1.22 (0.27)*^˄^	0.98 (0.23)*^ǂ^	0.76 (0.14)	1.13 (0.34)^˄^	1.06 (0.34)^˄^	0.84 (0.23)	0.73 (0.15)
**Fractal dimension M/L ( )**							
1.46 (0.06)^˄^	1.45 (0.06)	1.42 (0.07)*	1.44 (0.06)	1.45 (0.06)^˄^	1.45 (0.06)^˄^	1.38 (0.05)^ǂ^	1.41 (0.05)
**Fractal dimension A/P ( )**							
1.44 (0.06)^˄^	1.44 (0.05)	1.38 (0.05)	1.41 (0.06)	1.42 (0.07)	1.43 (0.07)	1.40 (0.05)^ǂ^	1.43 (0.05)

*^˄ǂ^Significant (p < 0.05) differences between: *groups, ^˄^surfaces, and ^ǂ^visual condition.

## Discussion

The purpose of this study was to compare postural control in girls with idiopathic scoliosis and their able-bodied counterparts. Three findings seem to have particular value for better understanding the specificity of erect stance regulation in adolescent scoliosis. First, the COP amplitude and speed related measures of postural steadiness revealed that AIS is not inferior to CON when it comes to performance in easy to medium difficult tasks, i.e. during normal unperturbed bipedal stance or with one sensory input undermined or eliminated. This indicates that AIS's postural stability is adequate for most daily activities, except for those that require the integration of all input signals along with the vestibular input. Second, the sway irregularity measured by the sample entropy was higher in AIS than in CON also in all tests but one, the most difficult of them. This implies that daily postural tasks are performed by AIS with less attentional involvement and/or increased automaticity and raises an intriguing question of the causes and consequences of using such a strategy. And third, the transfer from firm to foam surface resulted in entirely different changes in the COP complexity (fractality) in both groups. This result provides evidence that AIS's balance control shows asymmetry with respect to the vertical axis in comparison to CON. Identifying the causes of this asymmetry seems to be of importance in the treatment of scoliosis.

### Sway amplitude indices testify to the similarity of postural steadiness for AIS and CON in easier balance tests

Changes in COP radius indicate that the effects of sensory manipulation were dependent on the difficulty of postural task performed. In easy tasks, i.e. with proprioceptive deterioration by inserting a foam during EO trials or with visual deprivation while standing on a firm surface, similar effects were observed in both groups. This is confirmed by other authors^8,13^ though there are studies reporting inferior results in AIS even during relatively simple postural task^4^. Such discrepant results are not unexpected due to heterogeneity of subgroups with scoliosis^13,14^. On the other hand, supplementary sensory manipulations during EC trials or while standing on a foam surface resulted in a larger increase of the COP radius in the AIS than in the CON group which corroborates similar findings by Simoneau et al.^15^ or Haumont et al.^16^. This observation indicates a greater dependence of AIS on vision or proprioception^15^ as compared to control group when the proprioceptive or visual input, respectively, is altered.

In the same vein, the CON's better performance in the most difficult task probably indicates that they were supported by additional sensory information. It could only be a vestibular input because this system, due to its lower sensitivity, begins to take part in controlling the balance when the body sway exceeds the vestibular threshold^17^. However, unlike CON, AIS did not take advantage of this input, possibly considering it was not sufficiently reliable^6^. There is, of course, a competing explanation: AIS used this input, but it did not bring the expected results. Whatever it was, one can have serious doubts about either the reliability of the vestibular input or the excessive reliance on this system for the purpose of postural control in girls with AIS^18^. This is in line with reports by other authors^6,19,20^ who suggest that dysfunction of the vestibular system may be a contributing factor to the development of AIS.

Very similar observations arise from the results for the COP amplitude and velocity. However, in contrast to the COP radius, the latter two measures were computed in both planes of movement separately and indicated a similar deterioration of postural control in the ML and AP plane. It therefore seems that whatever the source of the postural control problems in our AIS group, they manifest equally in both planes. Possible differences between this result and other studies that indicate impaired postural steadiness of AIS in only one plane can also be attributed to the diversity of the subgroups.

Finally, there is one result from this study that appears counterintuitive as it points to an advantage of AIS over CON. Figure 2 shows the Surface × Vision × Group interaction for sway velocity, indicating the difference between groups during transfer from firm to foam surface. With eyes open AIS was evidently better than CON exhibiting much lesser increase in COP speed (p = 0.004) than CON. Since closing the eyes makes AIS lose that advantage to CON, it must be vision that plays a fundamental role in this transition and supports AIS in this task. Under these particular conditions, AIS either makes better use of its vision or pays less attention than CON to accomplishing this task.

### AIS exhibit lesser attentional involvement in postural regulation

An unexpected result regarding the specificity of postural control in AIS is presented in Fig. 3. It confirms the greater irregularity of the COP in the three easier trials, with the exception of the fourth which was the most difficult. This strictly corresponds to the changes in the COP amplitude and speed in both groups, where also only the fourth trial revealed a deteriorated balance in AIS. Closing the eyes on the firm surface resulted in a slight decrease in sway entropy in both groups, while on the foam surface the decrease was much greater, further revealing a Vision × Group interaction (p = 0.008). What could have caused these intergroup differences in sway regularity? The answer to this question should be sought in the specific adaptation of the AIS balance to their pathology. A similar line of reasoning in athletic population was proposed by Williams et al.^21^.

The reduced entropy with eyes closed may indicate a more conscious balance in these conditions which is in line with the research of other authors^22,23^. However, the much greater decrease in entropy after eyes closure on foam noted in the AIS group (Fig. 3, right panel) is puzzling^24^.

A reasonable explanation of these results assumes that the increased entropy of AIS in the three easier trials is a consequence of the specific experiences of these girls. This group, quite physically active, built their postural strategies in the presence of additional requirements, such as wearing a brace and performing exercises^9^. Adjusting to these additional difficulties caused the need to use more attentional resources, reducing their share in the balance itself. As a result, they increased postural automaticity in easier trials. Self-correcting exercises that strengthen the core and develop better posture awareness could also have a part here. An increase in postural automaticity has been shown after a single bout of core stability exercises in young women^25^. However, this strategy failed in the most difficult task for two reasons: no inter-task transfer and the lack of exercise under these conditions. It can be speculated that it was their advantage over CON in terms of higher postural control automaticity that allowed them to compete with CON in easier tasks.

### Complexity of sway suggests different between-plane asymmetry of postural control in AIS than in CON

Changes in postural strategies in both groups depending on the plane and support surface were presented in Fig. 4 illustrating the high interaction (p < 0.00079) of these factors on the COP fractal dimension. This interaction shows the intergroup differences in solving the problem of the transition from standing on the firm surface to the more difficult test on foam. Given the reasonable assumption that the control group was performing in an optimal way, this should be interpreted as their seeking for support (or stabilization) in the ML plane while allowing freedom of movement in the AP plane. It is worth recalling here that lower fractality indicates conservative actions aimed at safety, while its higher value means supporting adaptability to changing environmental requirements^26,27^. Such an alignment of postural correction targets to movement planes in healthy people seems reasonable and safer in more difficult postural tasks like standing on a foam. In case of a risk of falling, a stabilized body position in the ML plane with the predefined front and hind leg should speed up the step strategy to arrest the fall^28^. This is a simple mechanism that reduces the choice reaction time to a simple reaction time. Its essence is to increase the load on one of the legs, which automatically becomes the rear leg during the execution of the step strategy.

According to the current knowledge, a reduced fractality is also a symptom of unfavorable changes in the regulation of balance. This applies in particular to the reduction of the degrees of freedom, the limited adaptability to changing environmental demands and the less optimal use of afferent signals from the support surface^26,29^. Thus, the control group suffered the consequences of standing safely on a compliant ground, but only in the ML plane. As for the AIS group, traces of the same strategy are visible in its behavior with a decrease in the ML fractality by half as much as in CON. For some reason they have not been able to take full advantage of the potential importance of using the correct postural strategy in the ML axis. To make matters worse, they froze the degrees of freedom in the AP axis, which caused the body to stiffen in both planes. This gives the impression that the role of individual body axes in the regulation of balance is reversed, i.e. AIS display different between-plane asymmetry of postural control than CON.

Given the strange results of the fractal dimension in AIS on the foam, it seems important to look for possible causes. At the same time, it is worth remembering previous results of this study that indicated intergroup differences only during the most difficult task. The fractal dimension further extends these differences to standing on the foam with eyes open. This means that despite the seemingly correct performance on this task, the means used for its completion were disproportionate to the effect and revealed some deficiencies in AIS balance. Identifying the causes of these inadequacies should help address them and improve our approach to the disease.

The deterioration of proprioception only caused by standing on foam surface did not differentiate our two groups when we take into account the spatial measures of postural steadiness and sway entropy. It was only when they closed their eyes additionally that serious intergroup differences were revealed. However, in the case of sway fractality, the mere manipulation of proprioception was enough to detect these differences. In order to understand this increased sensitivity of fractal dimension, it is worth asking what difficulties appeared for the equilibrium system after both groups had switched to the foam surface. Firstly, the proprioceptive input has changed because both the contact forces under the feet and the ankle angles are completely different on the foam and firm surfaces. Secondly, for the same reason, the actuator had to perform less well, since it had to take into account the varying angles in the ankle joints and the changing forces under the feet in controlling the transmitted correction torques. And finally, the operation of the vestibular system was activated^17^ due to exceeding its threshold of sensitivity.

If the results in Fig. 4 were averaged for planes, even by visual inspection, it would turn out that the above-mentioned difficulties would cause an almost identical decrease in the value of the COP fractal dimension in both groups. Thus globally both groups share similar consequences of these sensorimotor challenges. However, such a transformation would hide very significant discrepancies between the groups in the individual planes, so its use would lead to wrong conclusions. These discrepancies irresistibly suggest a sort of interweaving of the functions of both planes in the performance of their sensory and/or control tasks. This seems to be in line with the Catanzariti et al.^30^ concept that AIS may be a consequence of an erroneous central vertical representation. In a similar vein, we have shown in our recent work^9^ that changes in internal representation of verticality may be associated with postural control improvement. In closing, it seems reasonable to speculate that errors in vertical perception may cause (or accompany) similar errors in AP or ML plane perception or, at least, the between-plane asymmetry in AIS that is different than in healthy peers. This certainly deserves further research.

The main limitation of this study is the lack of unambiguous explanation of the reasons for the observed intergroup differences. While some of our findings are entirely new, it is still unclear whether they were due to imperfect sensory integration, deficits in the vestibular system, or perhaps other sensorimotor disorders. This state of affairs reveals a certain helplessness of researchers in designing experiments that would more assuredly indicate what causes a deficit in postural control in AIS, let alone the factors leading to scoliosis. In order to understand well-hidden mechanisms for which we only observe the effects of their actions on the control of posture, it is necessary to conduct more focused research that will limit the number of possible alternatives.

## Data Availability

The datasets are available from the corresponding author on a reasonable request.
